# Non‐Immersive Virtual Reality Assessment of Functional Abilities in Older Adults is Less Influenced by Demographic Features than Traditional Cognitive Test Scores

**DOI:** 10.1002/alz70863_110620

**Published:** 2025-12-23

**Authors:** Moira McKniff, Marina Kaplan, Kimberly Halberstadter, Sophia L. Holmqvist, Molly B. Tassoni, Stephanie M. Simone, Riya Chaturvedi, Rachel Mis, Katherine Hackett, Tania Giovannetti

**Affiliations:** ^1^ Temple University, Philadelphia, PA USA; ^2^ The University of Texas at Austin, Austin, TX USA; ^3^ Icahn School of Medicine at Mount Sinai, New York, NY USA

## Abstract

**Background:**

Cognitive tests are crucial for clinical assessment of dementia, but are biased by education, sex, and race. We examined bias in a new measure of everyday cognition called the Virtual Kitchen Challenge (VKC), which requires participants to select objects and sequence task steps on a computer touchscreen to achieve highly familiar task goals (e.g., make breakfast/lunch). We hypothesized that because of its practical nature and high familiarity, scores from the VKC will not show demographic biases relative to conventional cognitive scores.

**Method:**

133 older adults (Mage= 73.40±6.53, range= 61‐98; 75% healthy cognition, 57% White, 36% Black, 63% female; education: M= 16.03±2.73) completed the VKC and cognitive tests from the Modified Preclinical Alzheimer's Cognitive Composite (mPACC; MMSE, HVLT‐D, BVMT‐D, Trail Making Test‐B, Animal Fluency). VKC scores (number of screen touches, percent of steps accomplished, time spent touching distractor objects, percent of time off‐screen) and a self‐report questionnaire (Functional Activity Questionnaire [FAQ]) were used to evaluate functional performance. Due to the small sample sizes of some racial groups, race analyses included only White and Black categories.

**Result:**

Better performance on the VKC measures was significantly associated with higher mPACC scores and lower FAQ rating (all *p* 's < .05). Black older adults performed significantly worse on mPACC tests (MMSE: *p* = .021, d= ‐.36; HVLT‐D: *p* <.001, d = ‐.52; BVMT‐D: *p* = <.001, d=‐.65; Trails B: *p* = .004, *d* = .44; Animal Fluency: *p* = .004, d=‐.44) compared to White older adults. These two groups did not significantly differ on any VKC measure (total touches *p* = .230; % accomplished *p* = .921; time with distractor objects *p* = .269; % time off‐screen *p* = .424). No significant sex differences were found in VKC or mPACC variables (all *p* 's > .05). Education was significantly correlated with all mPACC scores (*p* 's < .05) but not VKC (all *p* 's > .05).

**Conclusion:**

The VKC is not influenced by demographic features compared to traditional cognitive testing, underlining its potential importance as a more accurate assessment tool for cognitive decline. For this reason, it should be considered for clinical staging or as an outcome in clinical trials.

